# Relationship of strength, joint kinesthesia, and plantar tactile sensation to dynamic and static postural stability among patients with anterior cruciate ligament reconstruction

**DOI:** 10.3389/fphys.2023.1112708

**Published:** 2023-01-18

**Authors:** Shanshan Hu, Xiaoli Ma, Xiaoyuan Ma, Wei Sun, Zhipeng Zhou, Yan Chen, Qipeng Song

**Affiliations:** ^1^ College of Sports and Health, Shandong Sport University, Jinan, China; ^2^ Department of Orthopedic Surgery, Qilu Hospital, Cheeloo College of Medicine, Shandong University, Jinan, Shandong, China

**Keywords:** ACLR, body balance, muscle strength, rehabilitation, proprioception

## Abstract

**Objective:** Postural stability is essential for high-level physical activities after anterior cruciate ligament reconstruction (ACLR). This study was conducted to investigate the relationship of muscle strength, joint kinesthesia, and plantar tactile sensation to dynamic and static postural stability among patients with anterior cruciate ligament reconstruction.

**Methods:** Forty-four patients over 6 months post anterior cruciate ligament reconstruction (age: 27.9 ± 6.8 years, height: 181.7 ± 8.7 cm, weight: 80.6 ± 9.4 kg, postoperative duration: 10.3 ± 3.6 months) participated in this study. Their static and dynamic postural stability, muscle strength, hamstring/quadriceps ratio, joint kinesthesia, and plantar tactile sensation were measured. Partial correlations were used to determine the correlation of the above-mentioned variables with time to stabilization (TTS) and root mean square of the center of pressure (COP-RMS) in anterior-posterior (AP) and mediolateral (ML) directions.

**Results:** Both TTS_AP_ and TTS_ML_ were related to muscle strength and joint kinesthesia of knee flexion and extension; COP-RMS_AP_ was correlated with plantar tactile sensations at great toe and arch, while COP-RMS_ML_ was correlated with joint kinesthesia of knee flexion, and plantar tactile sensation at great toe and heel. Dynamic stability was sequentially correlated with strength and joint kinesthesia, while static stability was sequentially correlated with plantar tactile sensation and joint kinesthesia.

**Conclusion:** Among patients with anterior cruciate ligament reconstruction, strength is related to dynamic postural stability, joint kinesthesia is related to dynamic and static postural stability, and plantar tactile sensation is related to static postural stability. Strength has a higher level of relationship to dynamic stability than joint kinesthesia, and plantar tactile sensation has a higher level of relationship to static stability than joint kinesthesia.

## 1 Introduction

Anterior cruciate ligament (ACL) injury accounts for approximately 50% of all knee injuries (Kaeding et al., 2017). Approximately 80,000–250,000 ACL ruptures were reported each year in the United States (Cimino et al., 2010; Mall et al., 2014), and more than 500,000 ACL injuries were diagnosed in Europe annually (Bellitti et al., 2022). To restore the mechanical stability of the knee, about 80% of individuals with ACL rupture undertake anterior cruciate ligament reconstruction (ACLR) (Schilaty et al., 2017). In the past 20 years, the rate of ACLR has increased by over 60% (Dodwell et al., 2014). Although the physiological structure of the ligament can be repaired by ACLR, many patients still suffer from postural stability deficits after the surgery (Howells et al., 2011; Logerstedt et al., 2013).

Postural stability is essential for high-level physical activities after ACLR (Ortiz et al., 2014). The reduced postural stability delays the time to return to sports, severely shortening athletes’ careers and increasing their psychological burden (Clagg et al., 2015; Gokeler et al., 2017). Static standing is fundamental among many postures, and people who underwent ACLR have persistent deficits in static postural stability (Clark et al., 2014; Sugimoto et al., 2016). These deficits may explain, in part, the increased risk of future knee joint osteoarthritis and additional sport-related injuries (Sugimoto et al., 2016). Additionally, changes in dynamic postural stability can predict the likelihood of re-injury in ACLR patients (Paterno et al., 2010) and the deficit of postural stability increased the risk of injury on the reconstructed or non-reconstructed leg (Dauty et al., 2010). In turn, the re-injury further reduces postural stability, thus forming a pathological circulation (Fulton et al., 2014; Ortiz et al., 2014).

Significant postural stability deficits have been observed among patients with ACLR in both dynamic and static tasks (Colby et al., 1999; Webster and Gribble, 2010; Clark et al., 2014; Sugimoto et al., 2016). In laboratory and clinical practice, static postural stability is usually measured using the root mean square (RMS) of the center of pressure (COP) during standing, while dynamic postural stability is usually measured using the time to stabilization (TTS) during a dynamic task (e.g., single leg jump-landing) (Colby et al., 1999). It has been confirmed that compared to the controls, patients with ACLR have greater RMS of the COP (COP-RMS) (Goetschius et al., 2013; Stensdotter et al., 2016) and longer TTS (Colby et al., 1999; Webster and Gribble, 2010; Patterson and Delahunt, 2013).

Strength, joint kinesthesia, and plantar tactile sensation are three potential factors to maintain postural stability (Song et al., 2021). Joint kinesthesia is one of the proprioception senses, determined by establishing the threshold for detecting passive motion. When a perturbation occurs, signals from proprioceptive and tactile afferents evoke coordinated motor patterns, such as reflexes and automatic postural responses, which rapidly modify the locomotor pattern in response to perturbations (Frigon et al., 2021). These sensory afferents then convey sensory information to the central nervous system (Tahayori and Koceja, 2012) and finally cause muscle contraction that reflexively restores body stability (Wolfson et al., 1994; Gokeler et al., 2012; Song et al., 2021). Sufficient strength of the agonist and antagonist muscles across the joints is needed for good balance during functional activities (Croisier et al., 2008), and persistent muscle weakness may cause further alterations in the hamstring/quadriceps ratio resulting in dynamic instability, and it may increase the risk of further injury (Hohmann et al., 2019). Dysfunction of any part of the peripheral neural pathway may affect postural stability.

Compared to their peers, patients with ACLR has less strength (Schmitt et al., 2012), higher hamstring/quadriceps ratio (Kim et al., 2016), worse proprioception (Bonfim et al., 2003), and reduced plantar tactile sensation (Hoch et al., 2017). However, their relationship with postural stability is unclear. A significant correlation between postural stability and proprioception was detected in one study (Lee et al., 2009), but not in another (Ageberg et al., 2005). One study detected a significant correlation between postural stability with the strength of the quadriceps and hamstrings among patients with ACL injury (Ageberg et al., 2005), but no such correlation was detected in another study (Lee et al., 2009). Some studies indicated hamstring/quadriceps ratio has no relation with knee and body functions among patients with ACL tear (Lee et al., 2015) and ACLR (Hohmann et al., 2019), while some studies believed that dynamic balance depends on the strength balance among thigh muscle groups (White et al., 2003; Park et al., 2012). Additionally, to our knowledge, there is still a gap in the current works of literature regarding the relationship between postural stability and plantar tactile sensation among patients with ACL injuries. Certain rehabilitation methods have been shown to be effective in improving these three potential factors, for example, backward walking (Shen et al., 2019) or exergaming (Sadeghi et al., 2017) to improve joint motor sensation, swimming (Lee and Oh, 2015) or Nordic walking (Bullo et al., 2018) to increase strength, and Tai Chi (Zhang et al., 2021) to improve tactile sensation. Determining the relationship between strength, joint kinesthesia, and plantar tactile sensation with dynamic and static postural stability may help in selecting rehabilitation programs and facilitate the patients with ACLR to return to sports.

This study aimed to investigate the relationship of strength, joint kinesthesia, and plantar tactile sensation to postural stability among patients after ACLR. We hypothesized that 1) Strength, hamstring/quadriceps ratio, joint kinesthesia, and plantar tactile sensation are significantly related to static and dynamic postural stability as measured by the COP-RMS and the TTS. 2) Joint kinesthesia and strength contribute more to postural stability, compares with plantar tactile sensation.

## 2 Materials and methods

### 2.1 Participants

An *a priori* power analysis (G*Power Version 3.1) indicated that at least 27 participants are required to obtain an alpha level of 0.05 and a statistical power of 0.80 based on a previous report, which detected an r^2^ = 0.34 between proprioception and dynamic postural stability among 12 young (20–26 years) patients with ACLR (Lee et al., 2009). The participants were patients with high sports demand who underwent ACLR at a local hospital and rehabilitation center. The inclusion criteria were: 1) ages 18–40 years (Ma et al., 2022); 2) Tegner activity level ≥ 5 (Briggs et al., 2009); 3) regular participation in sports before the injury and willingness to return to sports after ACLR (Niederer et al., 2019); 4) unilateral ACL rupture and at least 6 months after ACLR (Ma et al., 2022); 5) absence of a history of neurological disease or vestibular or visual disturbance (Ma et al., 2022). The exclusion criteria were as follows: 1) associated knee ligamentous injuries within 3 months; 2) previous knee surgery; 3) clinically relevant cardiovascular history; 4) clinically relevant neuromuscular disorders; and 5) associated organ diseases that cannot be tolerated. A total of 44 participants were enrolled after the eligibility assessment (female = 14, male = 30, age: 27.9 ± 6.8 years, height: 181.7 ± 8.7 cm, weight: 80.6 ± 9.4 kg, BMI: 24.1 ± 3.6 kg/m^2^, postoperative duration: 10.3 ± 3.6 months) and included in the final analysis. Among them, 29 had ACLR with autologous hamstring tendons, 6 with autologous bone-patellar tendon-bone, 3 with allogeneic Achilles tendons, and 6 with artificial ligaments. All participants signed a written informed consent before participating in the study. Human participation was approved by the Institutional Review Boards of a local university (2022013) and was in accordance with the Declaration of Helsinki.

### 2.2 Protocol

The participants completed a battery of functional questionnaires, including the International Knee Documentation Committee questionnaire, Tegner, and Visual Analog Scale. The results were used to determine the participants for inclusion in the study. Dynamic and static postural stability, joint kinesthesia, and plantar tactile sensation were measured in a random order, while strength was measured last to avoid fatigue.

### 2.3 Static postural stability test

**FIGURE 1:**
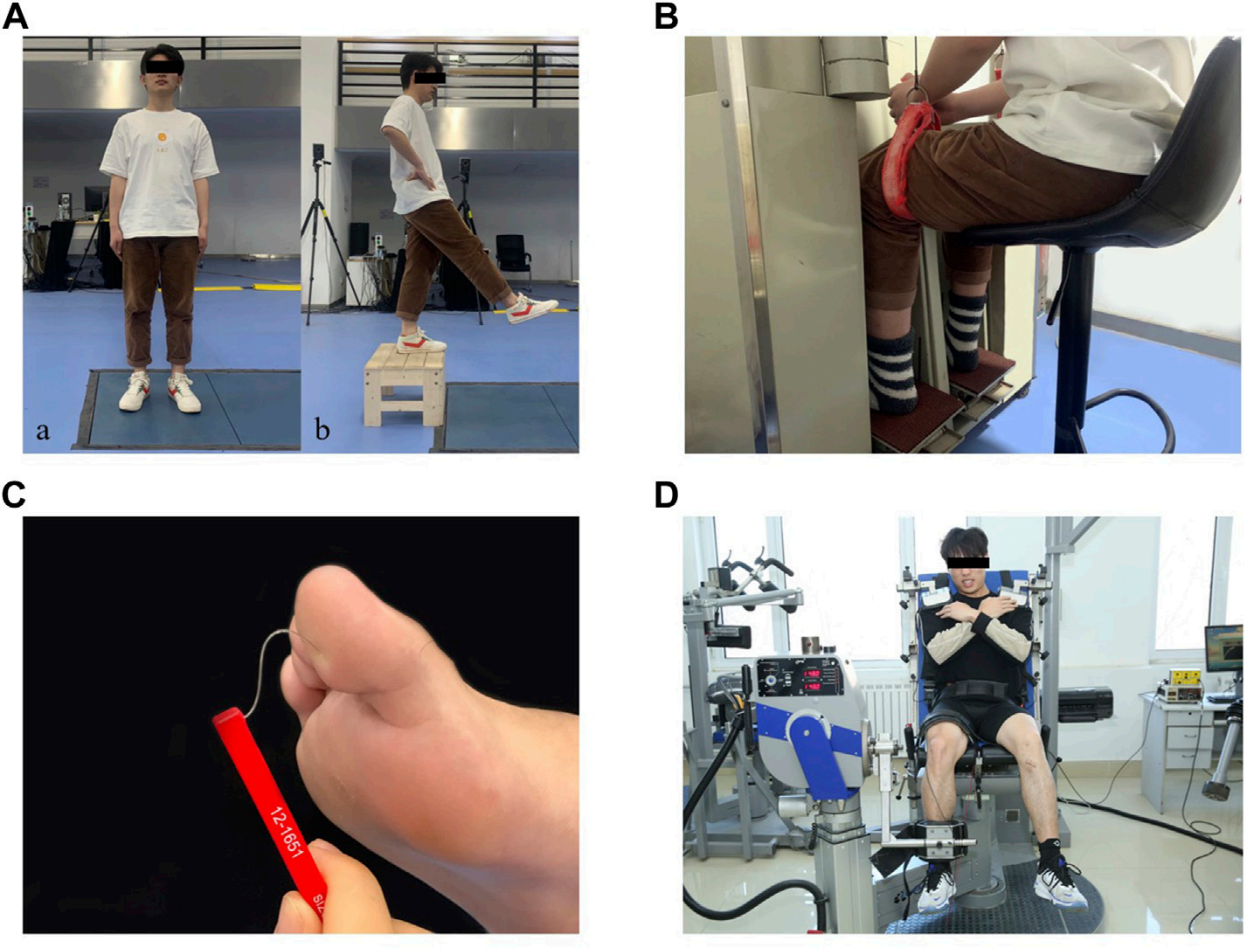
Test illustrations: **(Aa)**: Static postural stability test. **(Ab)**: Dynamic postural stability test. **(B)**: Joint kinesthesia test. **(C)**: Plantar tactile sensation test. **(D)**: Muscle strength test.

The participants stood upright on both legs with their eyes open on a force plate (AMTI, Inc., Watertown, MA, United States) for 120 s (Figure 1Aa), which showed good test-retest reliability when measuring COP variables [intraclass correlation coefficients (ICC) = 0.78–0.95] (Kouvelioti et al., 2015). They were instructed to look straight ahead with their feet at an angle of 14°, heels 17 cm apart, arms along the sides (McIlroy and Maki, 1997), and stand still during the test. Vertical ground reaction force (GRF) data between 60s and 90s of each trial were used in this study (Hatton et al., 2011). COP data were collected at a sampling rate of 1,000 Hz. Each participant performed three successful trials, and a successful trial was defined as maintaining balance for 120 s without any visible body movement. Each participant had at least a 1 min break between two consecutive trials. The mean value of the three trials were used for data analysis.

### 2.4 Dynamic postural stability test

Participants performed a jump-landing task to assess their dynamic postural stability (Figure 1Ab), which showed good test-retest reliability (ICC = 0.74–0.90) (Colby et al., 1999). They stood on top of a 35 cm high box in front of the force plate with their feet positioned shoulder width apart, hands at the waist, and looked straight ahead. Once the tester gave the command, participants stepped forward with their reconstructed legs and dropped from the box onto the force plate, and stand still on their reconstructed legs. They were instructed to stabilize as quickly as possible upon landing and to hold a still position for 20 s. Prior to the formal test, all subjects were allowed three practice trials for familiarization with the test procedure. GRF data were collected at a sample rate of 1,000 Hz. Three successful trials were recorded and at least 1 min of rest were given between jump-landing trials. A successful trial was defined as the participant landed without loss of balance or any visible corrections after initial contact (e.g., the other leg touching the ground, sliding the support limb). The mean value of the three trials were used for data analysis.

### 2.5 Joint kinesthesia test

The participants’ joint kinesthesia thresholds during knee flexion and extension of the reconstructed leg were assessed using a joint kinesthesia test device (Sunny, AP-II, China) (Figure 1B), which showed good test-retest reliability (ICC = 0.74–0.94) (Sun et al., 2015). The device consists of an operating platform and a test pedal. The platform was driven by two electric motors at an angular velocity of 0.4°/s. During the test, participants sat in an adjustable chair with their feet placed on the pedals, hips and knees flexed at 90° respectively, ankles in a neutral position, and lower legs perpendicular to the surface of the pedals. They wore blindfolds and headphones with music playing to eliminate visual and auditory stimuli from the testing environment. Participants were instructed to focus their attention on their reconstructed knee and to press the manual switch immediately to stop the movement of the pedal when they could sense the movement and identify the rotation direction. At the start of the trial, the motor was operated to rotate the knee to flexion or extension in a random sequence with random time intervals of 2–10 s. Each trial was started from the horizontal position of the platform. At least five trials were performed in each direction. The mean value of the minimum three angles sensed in each direction were used for data analysis.

### 2.6 Plantar tactile sensation test

The plantar tactile sensation of the reconstructed leg was assessed with a set of Semmes–Weinstein monofilaments (North Coast Medical, Inc., Morgan Hill, CA, United States) (Figure 1C), which showed good test-retest reliability (ICC = 0.83–0.86) (Collins et al., 2010). Six monofilaments with different sizes were used in this study: 2.83 (0.07 g), 3.61 (0.4 g), 4.31 (2 g), 4.56 (4 g), 5.07 (10 g), and 6.65 (300 g). Filament size = log10 (10 × force in milligrams). The filaments were applied randomly to the skin (bent 90°) on the bases of the great toe, first and fifth metatarsals, arch, and heel. A randomized null stimulus was added to ensure that participants could not anticipate the application of the filaments. Participants lay supine on the treatment table with their eyes closed and the tester selected filaments from thin to thick until they could perceive the stimulation and respond verbally to the correct location of the test area. The plantar tactile sensation threshold was determined by the thinnest monofilament they could feel (Feng et al., 2009).

### 2.7 Strength test

The strength of knee flexion and extension on the reconstructed leg was measured using a strength testing system (IsoMed 2000, D & R Ferstl GmbH, Germany) (Figure 1D), which showed good test-retest reliability in measuring lower limb strength (ICC = 0.77–0.98) (Gonosova et al., 2018). Participants were seated in a training chair with arms crossed in front of the chest, knees and hips at 90° and 85°, respectively, and secured with a lap belt across the thighs and pelvis and trunk. The lateral femoral condyle of the participant was aligned to the center of rotation of the dynamometer. Their isokinetic knee moments of flexion and extension were measured at 60°/s (Hoffman et al., 1999). Before testing, participants were asked to practice knee extension and flexion movements twice to ensure they understood how to exert force and complete the movements correctly. Once started, they were instructed to try their best to complete one knee extension and flexion movement. During the test, participants were encouraged to exert their maximum strength through verbal stimulation and visual feedback. Three trials were recorded, and at least a 2 min break was taken between two trials. Max knee flexion and extension torques were normalized by body mass (Nm/kg). The mean value of the three trials were used for data analysis.

### 2.8 Data extraction

**FIGURE 2:**
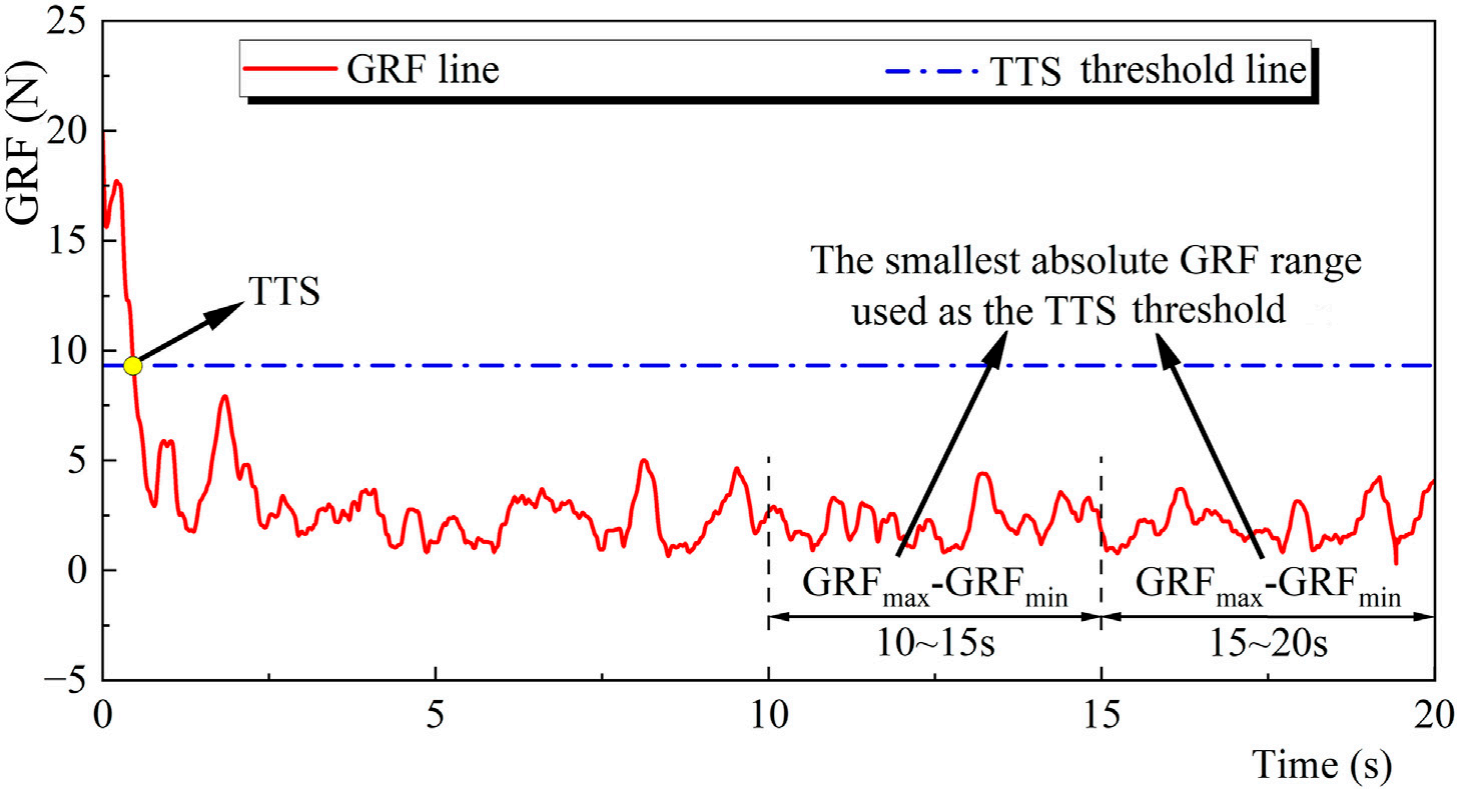
Illustration of the TTS calculation. Note: GRF, Ground reaction force; TTS, Time to stabilization.

Force plate data were used in calculating GRF and COP trajectory. COP was measured in the anterior-posterior (AP) and mediolateral (ML) directions. The GRF and COP data were filtered using a low-pass fourth-order Butterworth digital filter with a cut-off frequency of 50 Hz (Pan et al., 2016). The GRF data from the foot landed on the ground until the body regains stability were used in calculating the TTS (Figure 2). Two time-windows of the last 10 s (10–15, 15–20 s) of the AP and ML components of the GRF were analyzed. The windows with the smallest absolute GRF range for the AP and ML components were regarded as the optimal range of variation values (Ross and Guskiewicz, 2004). The 20 s COP data were collected from each participant after they landed on the ground.

### 2.9 Variables

The TTS was defined as the time from the foot landed on the ground until the body regains stability, i.e., the starting moment when the smoothed GRF was within the optimal range of variation values for at least 0.5 s (Tulloch et al., 2012).

The vertical ground reaction force data from 60 to 90 s during standing were collected (Hatton et al., 2011). The equations of RMS and mean velocity of the COP were as follows:

$$RMS\;of\;COP\;displacement\;\left(AP\right)=\sqrt{\frac{\sum {\left(x_{i}-\bar{x}\right)}^{2}}{N-1}}$$ (1)

$$RMS\;of\;COP\;displacement\;\left(ML\right)=\sqrt{\frac{\sum {\left(y_{i}-\bar{y}\right)}^{2}}{N-1}}$$ (2)

In these equations, ‾x and ‾y were the mean positions of CoP in the AP and ML directions.

The hamstring/quadriceps ratio was calculated by dividing the peak knee flexion torque by the peak knee extension torque in the strength test.

### 2.10 Data analysis

Descriptive analysis was used to summarize the means and standard deviations of the variables. The normality of all outcome variables was tested using Shapiro–Wilk test. A partial correlation (Pearson correlation for normally distributed or Spearman correlation for non-normally distributed data) was used to verify Hypothesis #1 by determining the correlations of the stability variables with each of the strength, joint kinesthesia, and plantar tactile sensation variables while controlling for covariates (gender, age, height, weight, and postoperative duration). Then, separate exploratory factor analysis was carried out among each category of the variables of interest. Multivariable linear regression was used to verify Hypothesis #2 by exploring the degrees of correlation between each generated factor and the stability variables while controlling for the above-mentioned covariates. The thresholds for the correlation coefficient (r) were as follows: 0–0.1, trivial; 0.1–0.3, weak; 0.3–0.5, moderate; >0.5, strong. All analyses were conducted in SAS 9.4, and the significance level was set at 0.05 (Cohen, 2013).

## 3 Results

Shapiro–Wilk tests showed that plantar tactile sensation data were non-normally distributed. Strength, joint kinesthesia, TTS, and COP-RMS data were normally distributed.

The descriptive characteristics are shown in Table 1. Mean, standard deviation, minimum value, and maximum value are reported for the TTS, COP-RMS, strength, joint kinesthesia, and plantar tactile sensation.

**TABLE 1:** Descriptive characteristics of outcome variables.

		Mean	SD	Max	Min
TTS (ms)	Anterior-posterior	843	250	1,377	451
	Mediolateral	544	302	1,428	83
COP-RMS (mm)	Anterior-posterior	0.84	0.52	2.89	0.18
	Mediolateral	0.93	0.59	2.70	0.28
Strength (N·m/kg)	Knee flexion	0.91	0.45	1.82	0.17
	Knee extension	1.21	0.56	2.93	0.44
	Flexion/extension ratio (%)	74.8	27.6	0.35	1.21
Joint kinesthesia (°)	Knee flexion	1.21	0.56	2.93	0.44
	Knee extension	0.96	0.54	2.49	0.22
Plantar tactile sensation (gauge)	Great toe	3.62	0.57	4.31	2.83
	1st metatarsal	3.71	0.41	4.31	2.83
	5th metatarsal	3.62	0.53	4.31	2.83
	Arch	3.78	0.35	4.31	2.83
	Heel	3.83	0.33	4.31	3.61

Abbreviations: TTS, time to stabilization; COP-RMS, root mean square of the center of pressure.

Partial correlations are shown in Table 2. Both TTS_AP_ and TTS_ML_ were related with strength and joint kinesthesia of knee flexion/extension; COP-RMS_AP_ was correlated with plantar tactile sensations at the great toe and arch, while COP-RMS_ML_ was correlated with joint kinesthesia of knee flexion and plantar tactile sensation at great toe and heel.

**TABLE 2:** Partial correlations of dynamic and static postural stability with strength, joint kinesthesia, and plantar tactile sensation.

Variables		Dynamic postural stability				Static postural stability			
		TTS_AP_		TTS_ML_		COP-RMS_AP_		COP-RMS_ML_	
		*r*	p	*r*	p	*r*	p	*r*	p
Strength (N·m/kg)	Knee flexion	−0.457	0.002	−0.419	0.005	−0.154	0.317	−0.117	0.448
	Knee extension	−0.446	0.002	−0.437	0.003	−0.056	0.720	0.098	0.525
	Flexion/extension ratio (%)	−0.246	0.108	−0.139	0.368	−0.198	0.198	−0.111	0.473
Joint kinesthesia (°)	Knee flexion	0.385	0.010	0.379	0.011	0.206	0.181	0.422	0.004
	Knee extension	0.393	0.008	0.539	<0.001	0.127	0.411	0.139	0.368
Plantar tactile sensation (gauge)	Great toe	0.270	0.077	0.060	0.699	0.503	<0.001	0.375	0.012
	1st metatarsal	0.107	0.487	0.126	0.014	0.290	0.056	0.198	0.199
	5th metatarsal	0.251	0.104	0.096	0.536	0.016	0.918	0.296	0.051
	Arch	0.280	0.855	0.013	0.931	0.371	0.013	0.194	0.207
	Heel	0.188	0.221	0.294	0.053	0.294	0.053	0.390	0.009

Notes: The correlations of plantar tactile sensation to stability were analyzed by Spearman correlation, others by Pearson correlation. The shaded cells represent significant correlation coefficients. The values were adjusted for gender, age, weight, height, and postoperative duration. Abbreviations: TTS_AP_, time to stabilization in the anterior-posterior direction; TTS_ML_, time to stabilization in the mediolateral direction; CoP-RMS_AP_, root mean square of the center of pressure in the anterior-posterior direction; CoP-RMS_ML_, root mean square of the center of pressure in the mediolateral direction; r, correlation coefficient.

The factor loadings for all the variables are shown in Table 3. Factor 1 (F1), factor 2 (F2), and factor 3 (F3) were the summaries of plantar tactile sensation, strength, and joint kinesthesia, respectively, with a Kaiser Meyer Olkin value of 0.718 and a sphericity of < 0.001.

**TABLE 3:** Factor loadings for the variables among the strength, joint kinesthesia, and plantar tactile sensation.

		Factor 1	Factor 2	Factor 3
Strength (N·m/kg)	Knee flexion	--	0.788	--
	Knee extension	--	0.863	--
Joint kinesthesia (°)	Knee flexion	--	--	0.973
	Knee extension	--	--	0.844
Plantar tactile sensation (gauge)	Great toe	0.837	--	--
	1st metatarsal	0.547	--	--
	5th metatarsal	0.640	--	--
	Arch	0.744	--	--
	Heel	0.608	--	--

Notes: --, factor loading < 0.500.

The equations for multivariable regression are:

$${TTS}_{AP}=843.30-114.30\times F2$$ (3)

$${TTS}_{ML}=554.07-94.63\times F2+159.45\times F3$$ (4)

$$COP-{RMS}_{AP}=0.84+0.24\times F1$$ (5)

$$COP-{RMS}_{ML}=0.93+0.23\times F1+0.36\times F3$$ (6)

In Eq. 3, adjusted *r* ^2^ = 0.249, p_F2_ = 0.001, and *β*_F2_ = 0.457; In Eq. 4, adjusted *r* ^2^ = 0.393, p_F2_ < 0.001, p_F3_ = 0.012, *β*_F2_ = 0.529, and *β*_F3_ = 0.314, *β*_F2_ > *β*_F3_ indicated strength contributed more to dynamic postural stability than proprioception. In Eq. 5, adjusted *r* ^2^ = 0.221, p_F1_ = 0.001, and *β*_F1_ = 0.465; In Eq. 6, adjusted *r* ^2^ = 0.479, p_F1_ < 0.001, p_F3_ = 0.002, *β*_F1_ = 0.602, *β*_F3_ = 0.390, *β*_F1_ > *β*_F3_ indicated plantar tactile sensation contributed more to static postural stability than proprioception.

## 4 Discussion

The main purpose of this study was to identify the relationship of dynamic and static postural stability to strength, joint kinesthesia, and plantar tactile sensation, and their degrees of contribution among patients with ACLR. The outcomes partly supported hypothesis #1 by indicating that strength is related to dynamic postural stability, joint kinesthesia is related to dynamic and static postural stability, and plantar tactile sensation is related to static postural stability, while rejected hypothesis #2 by indicating dynamic stability was sequentially correlated with strength and joint kinesthesia, and static stability was sequentially correlated with plantar tactile sensation and joint kinesthesia. The mean and range of results collected in this study were similar and comparable to previous studies investigating postural stability (Colby et al., 1999; Patterson and Delahunt, 2013), strength (Robinson et al., 2022), hamstring/quadriceps ratio (Hohmann et al., 2019), joint kinesthesia (Bonfim et al., 2003), and plantar tactile sensation (Hoch et al., 2017) among patients with ACL reconstruction.

Our results indicated that strength was related to dynamic but not to static postural stability. Previous studies supported our findings (Shiraishi et al., 1996; Kim et al., 2022). Some studies disagreed with our findings and indicated that strength was not related to dynamic postural stability (Novaretti et al., 2018) and was related to static postural stability (Cinar-Medeni et al., 2015), this may be caused by differences in testing protocols. Novaretti et al. (2018) measured the ability to maintain posture under dynamic stress on a circular platform, with up to 20° of tilting. Their protocol is more like a test of static postural control, rather than a dynamic one. Cinar-Medeni et al. (2015) used a single-leg stance task to assess static postural stability, and the movement of transitioning from standing on a double leg to being supported by a single leg may influence postural stability outcomes (Dingenen et al., 2015). The double-legged stance used in this study is considered a standard and reliable posture to assess static postural stability among patients with ACLR (Kouvelioti et al., 2015; Lion et al., 2018). During dynamic activities (e.g., cutting, pivoting, landing, etc.), the knee flexor and extensor contract synchronously to stabilize the knee and thus maintain postural stability (Redler et al., 2021). Compared with dynamic tasks, static standing requires less strength to maintain postural stability and relies more on plantar tactile sensation (Song et al., 2021). This study indicated that strength is correlated to dynamic stability, and our viewpoint is support by previous studies indicating that strength exercise benefits to the recovery of body stability among patients with ACLR (Shaw et al., 2005). Strength training should be employed to improve postural stability among patients with ACLR.

No significant correlations were detected between hamstring/quadriceps ratio and postural stability, agreed by previous studies indicating hamstring/quadriceps ratio has no relation with knee and body functions among patients with ACLR (Hohmann et al., 2019). One of the potential reasons is that with surgical reconstruction of the ACL, static knee stability is restored, and the need to downregulate quadriceps activity reduced with recovery. It has been pointed that a higher hamstring/quadriceps ratio represent a better ability of the hamstrings to stabilize the knee joint (Hiemstra et al., 2004). In our cohort, the hamstring/quadriceps ratio (74.8%) is higher than that among patients with ACL deficits (about 60%–66%) (Myer et al., 2009), agreed by a previous study indicating that patients has higher hamstring/quadriceps ratio after receiving ACLR (Jordan et al., 2015).

The results showed that joint kinesthesia was associated with both dynamic and static postural stability. Our observations were consistent with the previous studies (Borsa et al., 1998; Lee et al., 2009). One study disagreed with us by showing that proprioception was not related to either static or dynamic postural stability among patients with ACLR (Birmingham et al., 2001). They used joint position sense (JPS) to represent joint kinesthesia, unlike the joint kinesthesia in our study. JPS is more complex than joint kinesthesia (Reider et al., 2003). In JPS tests, participants must try to remember the position and then reproduce it accurately. We have reasons to infer that the correlation between JPS and postural stability may be influenced by the memory and learning effect of the participants. Previous studies supported our viewpoint by pointing out that joint kinesthesia is more reliable than position sense in detecting proprioceptive deficits in people with ACL injury or reconstruction (Fischer-Rasmussen and Jensen, 2000; Reider et al., 2003). This study showed that only knee flexion kinesthesia, but not knee extension kinesthesia, is associated with static stability. Knee flexion kinesthesia is provided by mechanoreceptors in the flexor muscles and flexor ligaments, and the ACL is considered agonists of the hamstrings with a knee flexion function (Hohmann et al., 2019). Therefore, it is reasonable to infer that mechanoreceptors in the ACL provide knee flexion kinesthesia. The impaired ACL among patients with ACLR makes the CNS dependent more on kinesthesia signals from the knee flexor muscles. This study further indicated that kinesthesia is correlated to dynamic and static stability, and our viewpoint is support by previous studies indicating that proprioceptive training appeared to decrease the incidence of injury to the knee and specifically the ACL (Cooper et al., 2005). Proprioceptive training should be employed among patients with ACLR.

Impaired postural stability in the ML direction is associated with a higher risk of falls compared to impaired postural stability in the AP direction (Islam et al., 2004). During movement, if the disturbance occurs in the AP direction, individuals can remove the disturbance by swinging their lower limbs to move their feet into the appropriate position. But if the disturbance occurs in the ML direction, it is difficult for them to drop their swing legs on the outside of the supporting leg to maintain postural stability. In addition, Chen et al. (Chen and Qu, 2019) found that ML postural stability deteriorated with decreased ankle proprioception. It is inferred that joint kinesthesia may be limited to ML direction in terms of maintaining postural stability after ACLR.

Our results showed that plantar tactile sensation was related to static postural stability but not dynamic postural stability. To our knowledge, the relationship between plantar tactile sensation and postural stability in the ACLR population has yet to be investigated. Previous findings in other populations were consistent with ours (Nyland et al., 1994; Song et al., 2021). During upright standing, the position of COP changes with slight variation (Meyer et al., 2004). The plantar cutaneous mechanoreceptors transmit spatial and temporal information concerning the pressure variations underfoot to the central nervous system (Kavounoudias et al., 1998), thus reflexively maintaining static postural stability. Unlike during standing still, muscles and tendons were stretched during dynamic locomotion, muscle spindle and Golgi tendon organ were activated, and proprioceptive signals provide movement and position information of body segments (Frigon et al., 2021). Type III sensory neurons, responsive to plantar tactile sensation, conduct slower and weaker signals than type Ia and type II sensory neurons, responsible for proprioception (Li et al., 2019). Therefore, patients with ACLR may rely more on joint kinesthesia rather than plantar tactile sensation to maintain dynamic postural stability.

Cutaneous sensitivity was partially influenced by the mechanical properties of the skin (Strzalkowski et al., 2015). The arch was the softest and thinnest site, followed by the great toe, fifth metatarsal and heel (Strzalkowski et al., 2015). Plantar sensitivity is correlated with plantar pressure distribution (Nurse and Nigg, 2001). The arch and great toe had better sensitivity, and significantly affected the plantar pressure distribution during standing, which were closely related to static postural stability (Zhang and Li, 2013; Song et al., 2021).

Multivariate linear regression showed that dynamic stability was sequentially correlated with strength at a higher level than joint kinesthesia, while static stability was correlated with plantar tactile sensation at a higher level than joint kinesthesia. It needs to be noted that although joint kinesthesia was related to both dynamic and static postural stability, it was outweighed by strength and plantar tactile sensation, respectively. It has been shown that the ACL contains mechanoreceptors (Schultz et al., 1984), which provide proprioceptive information about joint position and movement whilst coordinating muscular reflex stabilization of the knee joint (Mohammadirad et al., 2012). Once the ACL is ruptured, these sensory receptors are damaged, resulting in an altered sensory afferent information and disrupted neurofeedback circuit (Mohammadirad et al., 2012), which may decrease the level joint kinesthesia in relation to dynamic and static postural control.

There are some limitations to this study. First, the reconstruction objects selected by ACLR include autologous tendons, allogeneic tendons, and artificial ligaments. The impact of these three kinds of reconstruction objects on the postural stability of patients with ACLR may be slightly different. Further studies are recommended to subdivide each participant’s reconstruction objects and considered the potential influence of these reconstruction objects on postural stability among patients with ACLR. Second, only the effects of strength, joint kinesthesia, and plantar tactile sensation on postural stability among patients with ACLR were investigated in this study. Other factors, such as vision and vestibular system, might also have an impact on postural stability. Third, all the participants in this study were patients with high sports demand, so the findings may only apply to this population.

## 5 Conclusion

Among patients with ACLR, dynamic stability was sequentially correlated with strength and joint kinesthesia, while static stability was sequentially correlated with plantar tactile sensation and joint kinesthesia. Lower extremity strength training is vital to postural stability recovery since it is firstly related to dynamic stability, and sensations, both joint kinesthesia and plantar tactile sensation, should be emphasized since they related to both dynamic and static stability among patients with ACLR.

## Data Availability

The raw data supporting the conclusion of this article will be made available by the authors, without undue reservation.
